# Effect of a Cognitive Behavioral Therapy–Based AI Chatbot on Depression and Loneliness in Chinese University Students: Randomized Controlled Trial With Financial Stress Moderation

**DOI:** 10.2196/63806

**Published:** 2025-08-29

**Authors:** Yahui Wang, Xuhong Li, Qiaochu Zhang, Dannii Yeung, Yihan Wu

**Affiliations:** 1 Department of Social and Behavioural Sciences, City University of Hong Kong, Hong Kong, China; 2 Department of Sociology and Social Security, Ginling College, Nanjing Normal University, 1 Wenyuan Rd, Qixia District, Nanjing, Jiangsu, 210000, China, 86 13736333980

**Keywords:** chatbot, cognitive behavioral therapy, financial stress, university students, artificial intelligence, depression, loneliness, randomized controlled trial

## Abstract

**Background:**

Mental health concerns are prevalent among university students, with financial stress further compounding these issues. While cognitive behavioral therapy (CBT) is effective for these conditions, its delivery through artificial intelligence (AI) chatbots represents a promising approach, especially in non-Western contexts.

**Objective:**

This study aims to investigate the efficacy of a culturally adapted, CBT-based AI chatbot for improving the well-being of Chinese university students and to examine whether financial stress moderates its effectiveness.

**Methods:**

In this randomized controlled trial, 100 university students (mean age 20.8, SD 2.2 years; 62/100, 62% female) were allocated to either an intervention (n=50) or a waitlist control group (n=50). The intervention group interacted with a CBT-based AI chatbot for 7 consecutive days. Depression (Center for Epidemiologic Studies Depression Scale), anxiety (Generalized Anxiety Disorder-7 scale), and loneliness (UCLA Loneliness Scale) were assessed at baseline, day 3, and day 7. Financial stress was measured using the Psychological Inventory of Financial Scarcity.

**Results:**

Significant group×time interactions were found for depression (*F*_2,196_=8.63; *P*<.001; *η*²_p_=.08) and loneliness (*F*_2,196_=5.57; *P*=.004; *η*²_p_=.05), but not for anxiety (*F*_2,196_=1.31; *P*=.27; *η*²_p_=.01). Post hoc comparisons showed significant reductions in both depression (*t*=3.85; *P*<.001) and loneliness (*t*=4.28; *P*<.001) from baseline to postintervention in the intervention group, with corresponding effect sizes of Cohen *d*=0.71 (95% CI 0.30‐1.12) and Cohen *d*=0.60 (95% CI 0.20‐1.00), respectively. No significant changes were observed in the waitlist control group. Exploratory subgroup analyses revealed that participants with high financial stress demonstrated significantly greater improvements in depression (*F*_2,52_=11.56; *P*<.001; *η*²_p_=.31) and loneliness (*F*_2,52_=11.18; *P*<.001; *η*²_p_=.30) compared to those with low financial stress.

**Conclusions:**

The culturally adapted, CBT-based AI chatbot effectively reduced depression and loneliness in Chinese university students, with stronger effects among those experiencing high financial stress. These findings highlight the potential of AI-driven interventions to provide accessible mental health support, particularly for financially stressed students.

## Introduction

### Mental Health Challenges Among University Students and Financial Stress

University students face numerous stressors that can impact their mental health and well-being. Depression, anxiety, and loneliness are prevalent concerns in this population [1], with global estimates indicating that over 30% of tertiary students experience clinically significant symptoms [2]. Compared to primary and secondary school students, university students are at a higher risk of developing mental health issues due to the unique challenges they face, such as increased academic pressure, financial burden, and the transition to adulthood [3]. Existing studies consistently illustrate that anxiety and depression lead to a variety of negative outcomes among university students, such as poor academic performance, reduced social networks, low quality of life, and sleep problems [4,5].

In the postpandemic era, financial stress has significantly worsened the psychological health of university students in various countries, including the United States, United Kingdom, Germany, and China [6-8]. The rising costs of higher education, coupled with limited financial resources, place a significant burden on many students, leading to increased vulnerability to mental health problems [9]. Research consistently suggests that financial hardship is associated with higher levels of stress, anxiety, and depression among university students [10,11]. This relationship appears particularly pronounced in the postpandemic context, where economic uncertainties have intensified financial pressures on students [8]. Addressing the impact of financial stress on university students’ mental health is, therefore, increasingly crucial.

### Underuse of Mental Health Services

Despite the high prevalence of mental health issues, most university students seldom seek professional mental health services provided by their institutions or external providers [4,12]. A systematic review and meta-analysis study reported that 35% (95% CI 22%‐50%) of university students used mental health services, with individual study estimates ranging from 13.7% to 68.6% [13]. This substantial heterogeneity reflects differences in health care systems, cultural contexts, service definitions, and accessibility of services across countries and institutions. Notably, the same review also examined specific subgroups and service types, finding that overall outpatient use rates were particularly low among students in non-Western contexts, such as China (5.1%) and Bangladesh (7.1%), compared to the average rates in Western settings [13]. This East-West disparity highlights the particular challenges faced by students in Asian contexts.

The underuse of mental health services can be attributed to various barriers, such as stigma, time constraints, unaffordability, and limited accessibility [14-16]. These barriers are often more pronounced in collectivistic cultures like China, where mental health stigma is particularly strong and seeking help may be viewed as bringing shame to one’s family [17]. The combination of high need and low service use—especially pronounced in Chinese contexts—underscores the importance of developing innovative, culturally sensitive interventions that can overcome these substantial barriers to care.

### Digital Interventions and AI Chatbots as a Promising Solution

Digital mental health interventions, such as mobile apps and web-based self-help programs, show promise in overcoming treatment and access barriers [18]. These interventions can meet the high demand for campus counseling services and reduce access barriers by providing convenient, anonymous, and cost-effective support to students. A meta-analysis by Harrer et al [19] found that internet-based interventions were effective in reducing symptoms of depression, anxiety, and stress among university students, with effect sizes being comparable to those of face-to-face interventions. However, many digital interventions still face challenges with user engagement, personalization, and cultural relevance [20-22].

Recent advancements in artificial intelligence (AI) and natural language processing have paved the way for the development of AI-powered chatbots that can deliver psychological support through interactive conversations [23]. These AI-powered chatbots offer several advantages over traditional digital interventions: they provide 24/7 access to support, reduce stigma associated with help-seeking, and offer personalized, engaging experiences [24]. Emerging research supports their efficacy; for example, Fitzpatrick et al [25] found that a CBT-based conversational agent effectively reduced symptoms of depression and anxiety among young adults, while D’Alfonso [26] reported promising results using an AI chatbot for university students experiencing financial difficulties.

AI-powered chatbots can be particularly beneficial for university students, who are digital natives and may prefer seeking help through technology-based interventions [18]. The COVID-19 pandemic has further highlighted the importance of digital mental health interventions, as social distancing measures have limited access to in-person mental health services [27]. The pandemic has led to widespread social isolation and disruption of daily routines, which may exacerbate the risk of mental health problems among students [28]. Chatbots can provide a convenient and accessible source of support for students who may have smaller social networks or feel hesitant to share their problems with others due to the pandemic. Furthermore, the anonymous nature of chatbot interactions may encourage students who are hesitant to share their problems with parents, friends, or teachers to seek help [29]. Chatbot interventions can provide personalized support through interactive conversations, which may include psychoeducation, reflection prompts, in-session exercises, and personalized feedback based on users’ responses [25]. These interactive features can enhance user engagement, promote the acquisition and application of coping skills, and provide a sense of social support, which may be particularly valuable for students experiencing social isolation or loneliness [30].

### Cultural Adaptation of CBT: A Necessity, Not an Option

Cognitive-behavioral therapy (CBT) has demonstrated effectiveness for treating common mental health problems among university students [31]. However, cultural context significantly influences how mental health problems are experienced, expressed, and addressed, making cultural adaptation of psychological interventions not merely beneficial but essential for maximizing effectiveness.

The need for cultural adaptation is particularly clear in Chinese contexts, where mental health experiences differ markedly from Western norms [17]. In Chinese culture, mental health problems are often viewed through a collectivistic lens where individual psychological issues can threaten family reputation (“face”), creating a “courtesy stigma” that extends beyond the individual to their entire family unit, which can intensify stigma and discourage help-seeking [32]. This family-oriented stigma creates stronger barriers to help-seeking compared to more individualistic Western societies. In addition, traditional Chinese values emphasize emotional restraint and self-reliance and the importance of maintaining social harmony, which may conflict with Western therapeutic approaches that encourage emotional expression and seeking external help [33]. Mental health issues in Chinese contexts are often conceptualized in somatic terms rather than psychological ones, with cultural idioms of distress frequently manifesting as physical complaints [34].

These cultural differences are not merely theoretical but have demonstrated practical implications for intervention effectiveness. Several studies have found that standard Western CBT interventions may have lower engagement and effectiveness when applied directly to Chinese populations without cultural adaptation [35,36]. For example, Hwang et al [36] demonstrated that culturally adapted CBT for Chinese Americans showed not only significantly better clinical outcomes but also substantially lower dropout rates compared to standard CBT. Similarly, Choi et al [37] found that cultural adaptation of CBT significantly improved treatment adherence and outcomes among Chinese Australians.

A recent meta-analysis by Li et al [38] examining culturally adapted interventions for common mental disorders in people of Chinese descent found these adaptations beneficial, though the efficacy did not differ significantly between culturally modified interventions and culturally specific interventions. Rathod et al [39] also reviewed culturally adapted psychological interventions and found varying levels of effectiveness across different cultural groups and adaptation approaches. These highlight that thoughtful adaptation, whether modifying existing approaches or developing culturally specific ones, can improve outcomes when grounded in an understanding of core cultural concepts and values while maintaining essential therapeutic mechanisms.

### Research Gaps and This Study

Despite the promising potential of AI-powered chatbots and the importance of cultural adaptation, significant research gaps remain. First, most studies evaluating digital mental health interventions have been conducted with Western samples, limiting generalizability to Chinese university students [40,41]. Second, as revealed by a systematic review of digital mental health in China [42], Chinese studies rarely adopt co-production approaches in developing digital health technologies and often demonstrate less rigorous methodological quality. Third, few studies have examined whether digital mental health interventions are equally effective across different levels of financial stress, which is an increasingly important consideration given growing economic pressures on students.

Most critically, despite the theoretical promise of combining AI chatbot technology with culturally adapted CBT content, very few randomized controlled trials have evaluated such interventions specifically for Chinese university students experiencing financial stress. This represents a significant missed opportunity to address an urgent public health need through innovative, scalable approaches that could potentially overcome cultural and structural barriers to care.

This study addresses these gaps by evaluating the efficacy of a custom-developed, culturally adapted, CBT-based AI chatbot in reducing depression, anxiety, and loneliness among Chinese university students, with particular attention to the moderating role of financial stress. Using a randomized controlled trial design, we tested two primary hypotheses:

- Hypothesis 1: Students who engaged with the culturally adapted chatbot would show greater improvements in depression, anxiety, and loneliness compared to those in the waitlist control group.
- Hypothesis 2: The intervention’s effectiveness would be moderated by participants’ baseline levels of financial stress, with those experiencing higher financial stress benefiting more from the intervention compared to those with lower financial stress.

By examining both the overall efficacy of the chatbot and the moderating effect of financial stress, this study aims to provide valuable insights into how AI-driven, culturally adapted interventions might be optimally deployed to improve the well-being of vulnerable student populations.

## Methods

### Study Design and Participants

This study used a parallel-group, randomized controlled trial design. Participants were recruited from multiple public universities through email advertisements and campus flyers. The inclusion criteria were (1) being a currently enrolled full-time undergraduate or graduate student aged 18‐24 years, (2) owning an Android smartphone, and (3) being willing to engage with the chatbot daily for 7 days. The exclusion criteria included (1) current engagement in any type of psychotherapy, (2) having a diagnosis of any severe mental illness in history (eg, schizophrenia, bipolar disorder, etc), and (3) current use of psychiatric medications. Initial screening was conducted via online questionnaire (Wenjuanxing), and eligible participants were invited for further screening interviews conducted by trained research staff to confirm eligibility and obtain informed consent.

### Ethical Considerations

The study received ethical approval from the Institutional Review Board of Nanjing Normal University (NNU202301018). All participants provided written informed consent before enrollment. The consent process explicitly detailed the study purpose, procedures, potential risks and benefits, data handling protocols, and participants’ right to withdraw at any time without penalty. Data were anonymized using participant identification codes, and all information was stored on encrypted, password-protected servers compliant with institutional data security standards. Participants who completed the study received monetary compensation for their time and contribution. The study was registered with the Chinese Clinical Trial Registry (ChiCTR2500100868).

### Sample Size Determination and Randomization

A priori power analysis using G*Power 3.1 (Heinrich-Heine-Universität Düsseldorf) was conducted to determine the necessary sample size for detecting a small effect size (Cohen *f*=0.2) with 80% power at a significance level of *α*=.05. The anticipated effect size was based on meta-analytic findings by Firth et al [43], who reported effect sizes for smartphone-based mental health interventions targeting depression ranging from *g*=0.22 to *g*=0.56, depending on control condition type. We selected *f*=0.2 as a conservative estimate appropriate for our brief 7-day intervention with waitlist control, representing a clinically meaningful effect size within the empirically derived range.

The analysis was based on a 2 (group: intervention vs control) × 3 (time: T1, T2, T3) mixed ANOVA design, considering the interaction between within-subjects and between-subjects factors. The power analysis indicated that a total sample size of 42 participants (21 per group) was required. Adjusting for an anticipated 31% attrition rate (the weighted average for internet-based treatment programs according to a systematic review by Melville et al [44]), the adjusted total sample size was 60. However, to ensure sufficient power and account for potential dropouts, we aimed for a larger sample of 100 participants, which would maintain adequate statistical power even with higher-than-expected attrition. Detailed power analysis parameters and post hoc calculations for moderation analyses are provided in Multimedia Appendix 1.

A total of 120 students were recruited, of which 100 met the inclusion criteria and were enrolled in the study. Participants were then randomly allocated in a 1:1 ratio to either the intervention group (n=50) or the waitlist control group (n=50) using a computer-generated block randomization sequence created by an independent statistician not involved in recruitment. Group allocation was concealed through a digital randomization system managed by a researcher not involved in participant recruitment or assessment. The system revealed allocations only after participants completed the baseline assessments, ensuring that neither participants nor researchers could predict group assignments.

### Intervention

#### CBT-Based AI Chatbot Development

The CBT-based AI chatbot “Psy-Bot” was developed through a systematic, multistage process by our interdisciplinary research team consisting of clinical psychologists, AI experts, cultural adaptation specialists, and user experience designers. The development followed established frameworks for digital mental health interventions [45] and cultural adaptation of psychological treatments [36,46,47].

#### Content Development and Theoretical Foundation

The content was grounded in empirically supported CBT principles [48], with specific adaptation for university students and the Chinese cultural context. The development process included 3 key phases.

First, a comprehensive review of CBT protocols effective for depression, anxiety, and loneliness in university students was conducted, with particular focus on studies concerning financial stress. Key therapeutic components were identified from meta-analyses [49] and clinical practice guidelines.

Second, core therapeutic components were implemented based on Beck’s cognitive model [48] and included cognitive restructuring (identifying and challenging negative automatic thoughts), behavioral activation (scheduling pleasant activities and reducing avoidance), problem-solving training (systematic approach to addressing stressors), mindfulness exercises (present-focused awareness training), and social skills development (communication and assertiveness training).

Third, content was systematically adapted using Bernal’s framework for cultural adaptation [46], which addresses 8 dimensions: language, persons, metaphors, content, concepts, goals, methods, and context. This process involved consultations with experts in Chinese mental health (n=3) and focus groups with Chinese university students (n=10), review by bilingual psychologists to ensure linguistic and conceptual equivalence, and pilot-testing with the target population.

#### The 7-Day Module Structure

The chatbot content was structured as a progressive seven-day CBT-based intervention, with each daily module building on previously learned skills:

Day 1: Introduction to CBT principles established the cognitive-behavioral model and foundational skills for identifying thoughts, emotions, and behaviors.

**Figure 1.**
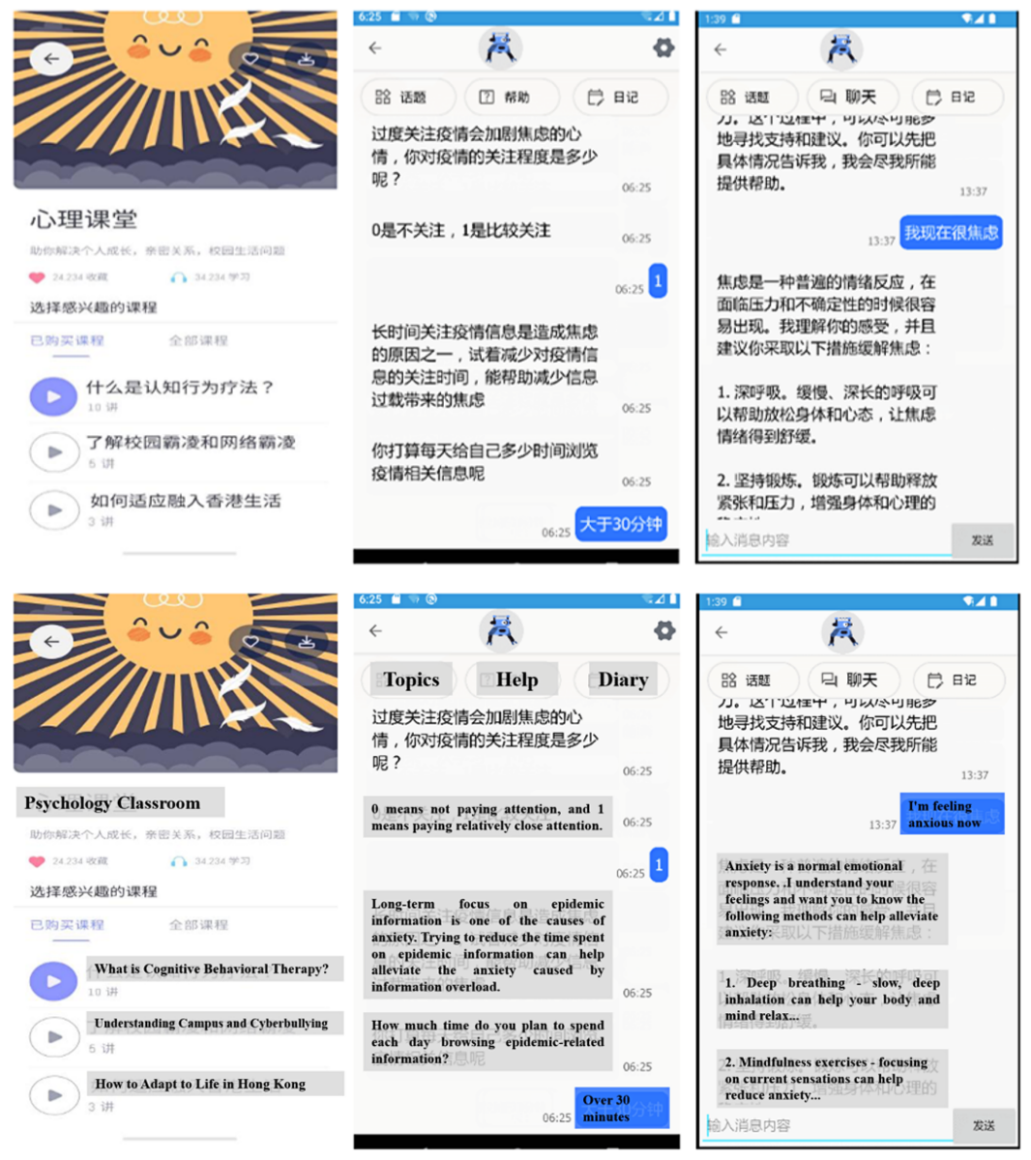
App interface screenshots with English translations. (**A**) Module selection: The app provided various modules, including an introduction to CBT, coping with campus bullying, and adapting to new environments. (**B**) Chat interface: Users could select a topic, initiate a conversation with the chatbot, and access a personal diary feature. The displayed dialog demonstrates how the chatbot helps users relieve anxiety by explaining its nature and providing coping methods. (**C**) Pandemic-related anxiety: This section features an interactive conversation about pandemic-related anxiety, where the chatbot provides personalized responses and guidance based on the user’s input. Chinese interface elements are shown with English translations below. CBT: cognitive behavioral therapy.

Day 2: Coping with campus-related stressors addressed interpersonal challenges including campus bullying (as shown in Figure 1A), using cognitive restructuring and assertiveness techniques.

Day 3: Adapting to new environments helped students navigate transitions and adjustment difficulties (specifically displayed in the app interface; see Figure 1A), using behavioral experiments and gradual exposure.

Day 4: Managing anxiety incorporated both general anxiety management and specific contexts such as pandemic-related anxiety (as shown in Figure 1C), using cognitive reframing and relaxation strategies.

Day 5: Financial stress management addressed cognitive patterns related to financial scarcity, problem-solving for financial challenges, and adaptive coping strategies, developed based on research on financial stress interventions.

Day 6: Emotion regulation and mood improvement focused on behavioral activation and mindfulness exercises to address depressive symptoms and enhance positive emotions.

Day 7: Building resilience and relapse prevention, consolidated learning and established maintenance strategies for continued well-being after the intervention period.

Each module was designed to be completed in approximately 20‐30 minutes. The complete module content and structure are presented in Table S1 in Multimedia Appendix 2.

#### Cultural Adaptation

As noted above, all content was systematically adapted following Bernal et al’s [46] empirically validated framework for cultural adaptation. This framework has been successfully applied in previous cultural adaptations of psychological interventions for Asian populations [35,36].

Key cultural adaptations included using indirect communication patterns aligned with Chinese preferences for emotional expression, incorporating collectivistic values emphasizing family harmony, addressing “face” concerns related to seeking help and financial difficulties, and integrating culturally resonant metaphors and Chinese idioms. Expert ratings indicated strong cultural appropriateness (mean rating 4.1/5 for cultural relevance). Detailed examples of cultural adaptations across all 8 dimensions of Bernal and colleague’s [46] framework are provided in Table S2 in Multimedia Appendix 2.

#### Technical Architecture and User Experience

The chatbot used a hybrid approach to response generation, combining rule-based responses with natural language processing to ensure clinical validity and safety. The system architecture included a dialog management system based on the RASA open-source framework (Rasa Technologies Inc) [50], with custom extensions for therapeutic dialog; natural language understanding implemented using bidirectional encoder representations from transformers–based models [51], fine-tuned on therapeutic conversations in Chinese; response generation combining pre-scripted therapeutic responses (70%), template-based dynamic responses (20%), and contextually generated responses (10%); and safety monitoring with automated detection of severe distress or risk expressions.

As shown in Figure 1A, the app interface allowed users to browse modules, mark favorites, and track popularity. The chatbot delivered content through conversational interactions (see Figure 1B), explaining psychological concepts, providing coping strategies, and engaging users in interactive therapeutic exercises. For specific concerns like pandemic-related anxiety, the chatbot provided tailored guidance based on individual expressions of distress (see Figure 1C).

The app incorporated gamification elements, such as progress tracking and virtual rewards, to enhance user engagement. It also sent gentle reminders if a session was missed. User interactions, including session completion and duration, were automatically logged in a secure Firebase database.

#### Validation Process

The chatbot underwent a rigorous 3-stage validation process:

1. Content validation: A total of 3 experts in CBT, digital mental health, and Chinese cultural psychology independently rated each module using standardized assessment forms. Mean ratings showed strong adherence to CBT principles (mean 4.2, SD 0.15, out of 5), cultural relevance (mean 4.1, SD 0.14, out of 5), and anticipated engagement (mean 3.9, SD 0.28, out of 5).
2. Technical validation: Comprehensive testing of system functionality, response accuracy, data security, and user experience was conducted by both technical staff and independent testers. This included simulating dialog turns and stress-testing the system under various input conditions.
3. Pilot-testing: A preliminary pilot test with 10 university students (separate from the main study sample) was conducted to gather feedback through structured questionnaires. The System Usability Scale [52] yielded a mean score of 82.3 (SD 3.83) out of 100 (range 76‐89), indicating excellent usability according to established benchmarks [53]. User experience was further assessed using a modified version of the Mobile Application Rating Scale [54], which showed good comprehension of therapeutic content (mean rating 4.4, SD 0.20, out of 5; range 3.9‐4.7).

Based on validation feedback, refinements were made to the interface and conversational flow before the main trial. Detailed validation metrics are presented in Table S3 in Multimedia Appendix 2, and sample dialog exchanges demonstrating the application of CBT techniques with cultural adaptations are provided in Table S4 in Multimedia Appendix 2.

#### Module Delivery and User Interaction

Participants in the intervention group accessed the chatbot through a mobile app installed on their Android smartphones via an APK file. The app featured a user-friendly interface with a chat window, notification settings, and progress tracking (see Figure 1). The chatbot delivered the modules through a conversational interface, using a combination of predefined scripts and dynamic response generation. It also incorporated multimedia elements, such as images and audio clips, to enhance user engagement.

The app was designed to unlock 1 module per day to ensure that participants followed the intended learning schedule. Participants were encouraged to complete 1 module per day, with the app sending gentle reminders if a session was missed. All materials used in the chatbot were either original content developed by the research team or sourced from public domain resources with proper attribution.

#### Waitlist Control Group

Participants in the waitlist control group did not receive access to the chatbot during the 7-day study period. They were informed that they would receive access to the app after completing the postintervention assessment. To minimize attrition, the waitlist control group participants received daily check-in messages expressing appreciation for their continued participation and reminding them of the upcoming assessments. The participants in the waitlist control group were asked to complete the same set of questionnaires as the intervention group at the 3 time points of assessment (baseline, midintervention, and postintervention).

#### Measures

Online self-report questionnaires were administered at 3 time points: baseline (T1, before the intervention on day 1), midintervention at day 3 (T2, day 3), and postintervention at day 7 (T3, day 7). The primary outcomes were as follows:

- Depression: Measured using the Chinese version of the 10-item Center for Epidemiologic Studies Depression Scale (CES-D-10) [55], a 10-item scale assessing the frequency of depressive symptoms over the past week on a 4-point Likert scale. Total scores range from 0 to 30, with higher scores indicating more severe depressive symptoms. In this study, the Cronbach α for the CES-D-10 were 0.89, 0.89, and 0.91 at T1, T2, and T3, respectively.
- Anxiety: Assessed using the Generalized Anxiety Disorder-7 scale (GAD-7) [56]), a 7-item measure of anxiety symptoms over the past 2 weeks, rated on a 4-point Likert scale. Higher scores indicate greater levels of anxiety (range: 0‐21). In this study, the Cronbach α was 0.92 at T1, 0.93 at T2, and 0.94 at T3.
- Loneliness: Measured using the UCLA Loneliness Scale (version 3) [57], a 20-item scale assessing subjective feelings of loneliness and social isolation on a 4-point Likert scale. Higher scores indicate greater loneliness (range: 20‐80). In this study, the Cronbach α was 0.93 at T1, 0.94 at T2, and 0.95 at T3.
- Financial stress: Participants also completed the Psychological Inventory of Financial Scarcity (PIFS) [58] at baseline, which is a 12-item scale measuring subjective perceptions of economic hardship on a 7-point Likert scale. Higher scores indicate greater financial stress. In this study, Cronbach *α* was 0.93 at T1, 0.94 at T2, and 0.95 at T3.

### Statistical Analysis

Quantitative data were analyzed using R software (version 4.3.0; R Core Team). Before the main analyses, data were screened for outliers and violations of statistical assumptions, with no significant issues detected. Our screening process involved visual inspection of boxplots and calculation of *z* scores, with values exceeding ±3.29 considered potential outliers. No extreme outliers requiring special handling were identified in the dataset. Missing data (attrition rate 11%, 11/100) were handled using the last observation carried forward (LOCF) method, maintaining the intention-to-treat principle while considering the study’s short duration (7 d).

Baseline group differences in demographic characteristics and outcome measures were examined using independent *t* tests for continuous variables and *χ*^2^ tests for categorical variables. To assess intervention effects, 2 (group: intervention vs control) × 3 (time: T1, T2, T3) mixed ANOVAs were conducted for each outcome variable, with group as the between-subjects factor and time as the within-subjects factor. Significant group×time interactions were probed with pairwise comparisons using Bonferroni correction to control for multiple testing. Effect sizes were reported using partial eta-squared (*η*²_p_) to provide a measure of the magnitude of the intervention effects. For outcomes with significant group×time interactions, Bonferroni-corrected pairwise comparisons were conducted to identify specific patterns of change. In addition, Cohen *d* effect sizes were calculated to quantify the magnitude of within-group changes from baseline to postintervention (T1 to T3) and between-group differences in change scores.

To examine the potential moderating role of financial stress on the intervention’s effectiveness, a moderation analysis was first conducted on the entire sample using mixed linear models. Exploratory subgroup analyses were conducted to further examine potential differential intervention effects based on participants’ baseline financial stress levels. The sample was median-split into high and low financial stress subgroups based on baseline PIFS scores. Repeated measures ANOVAs were then conducted separately for each subgroup and outcome variable, with time as the within-subjects factor. This approach allowed us to examine whether the intervention had differential effects depending on participants’ level of financial stress.

## Results

### Participant Characteristics

**Figure 2.**
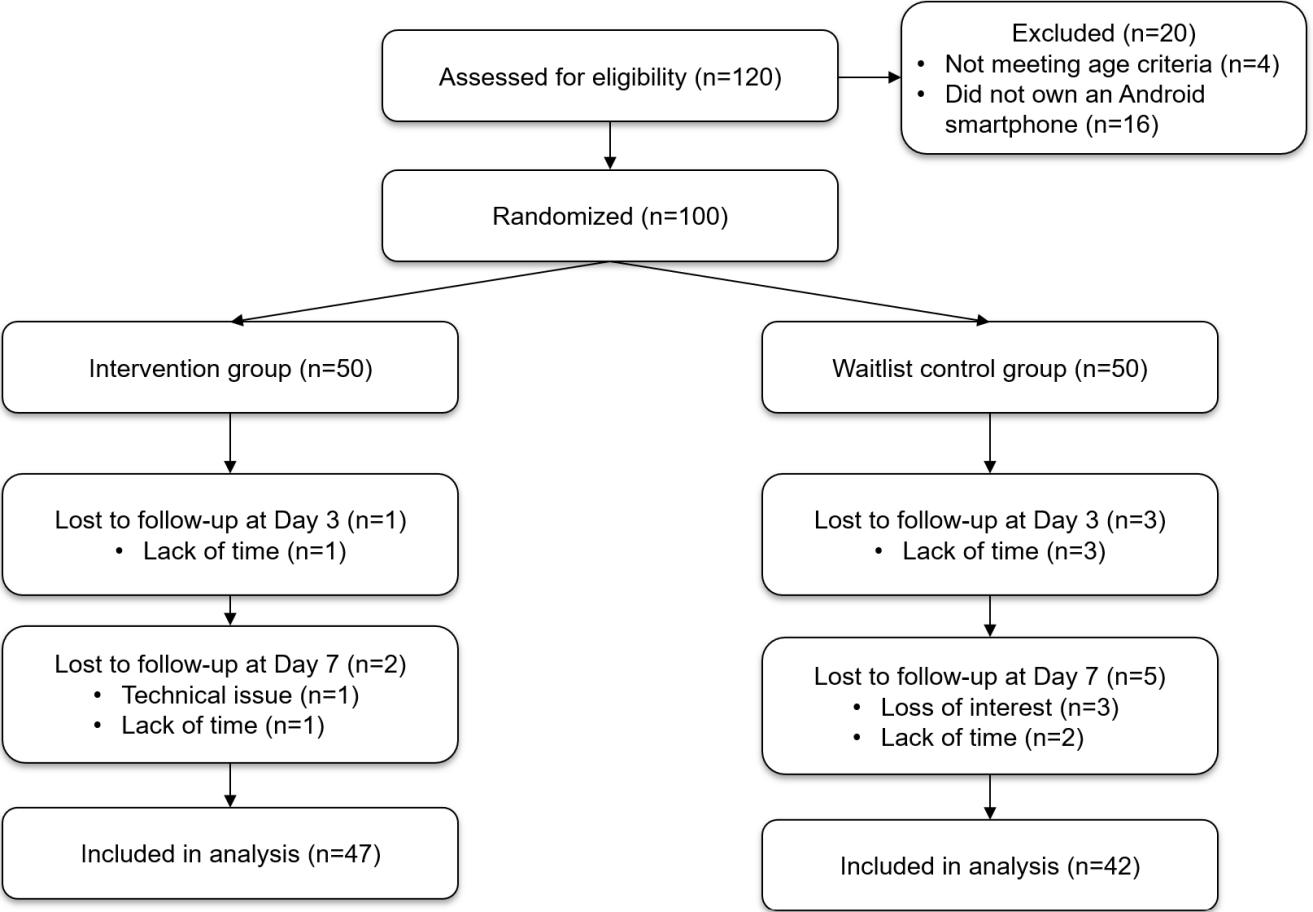
The CONSORT flow diagram. CONSORT: Consolidated Standards of Reporting Trials.

The CONSORT (Consolidated Standards of Reporting Trials) flow diagram (Figure 2) illustrates the participant flow through each stage of the study.

The final sample comprised 100 university students (mean age 20.8, SD 2.24 y; 62/100, 62% female) who were included in the intention-to-treat analysis using LOCF imputation. There were no significant baseline differences between the intervention and waitlist control groups on any demographic or outcome measures (*P*>.05). Detailed sample characteristics are presented in Table 1.

Baseline group differences were examined using independent 1-tailed *t* tests for continuous variables (age, CES-D, GAD-7, UCLA Loneliness Scale, and PIFS) and *χ*^2^ tests for categorical variables (sex). There were no significant differences in age (*t*_80.91_=0.80; *P*=.43), sex (*χ*^2^_1_=0.04; *P*=.84), CES-D scores (*t*_97.81_=0.59; *P*=.56), GAD-7 scores (*t*_98.00_=0.98; *P*=.33), or UCLA Loneliness scores (*t*_97.88_=1.09; *P*=.28) between the intervention and waitlist control groups at baseline. Regarding financial circumstances, there was a marginally significant difference between groups in PIFS scores (*t*_96.05_=1.81; *P*=.073), with the intervention group reporting slightly higher financial stress (mean 49.60, SD 14.67) than the control group (mean 44.10, SD 16.24), though this difference did not reach statistical significance at the conventional alpha level.

**Table 1.** Baseline demographic characteristics of participants (N=100).

Variable	Intervention (n=50)	Control (n=50)	Test statistic		*P* value
			*t* test (*df*)	Chi-square (*df*)	
Age, mean (SD)	20.96 (2.72)	20.60 (1.65)	0.80 (80.91)	—^a^	.43
Sex, n (%)			—	0.04 (1.00)	.84
Female	32 (64)	30 (60)			
Male	18 (36)	20 (40)			
CES-D^b^, mean (SD)	14.00 (5.98)	13.28 (6.25)	0.59 (97.81)	—	.56
GAD-7^c^, mean (SD)	13.62 (4.91)	12.66 (4.92)	0.98 (98.00)	—	.33
UCLA^d^, mean (SD)	47.14(11.67)	44.64 (11.26)	1.09 (97.88)	—	.28
PIFS^e^, mean (SD)	49.60 (14.67)	44.10 (16.24)	1.81 (96.05)	—	.07

a Not available.

b CES-D: Center for Epidemiologic Studies Depression Scale.

c GAD-7: Generalized Anxiety Disorder-7.

d UCLA: UCLA Loneliness Scale.

e PIFS: Psychological Inventory of Financial Scarcity Scale.

### Attrition and Engagement

The overall attrition rate was 11% (11/100), with no significant difference between the intervention (6%, 3/50) and control (16%, 8/50) groups. In the intervention group, 47 participants completed all seven sessions, while 3 participants did not fully complete the intervention. In the waitlist control group, 3 participants did not complete the midintervention assessment on Day 3, and 5 participants did not provide valid responses to the postintervention assessment on Day 7. Reasons for dropout included lack of time, loss of interest, and technical difficulties with the app.

### Intervention Effects on Mental Health Outcomes

Mixed-design ANOVAs were conducted to assess the intervention effects on each outcome variable (CES-D, GAD-7, and UCLA Loneliness Scale), with group (intervention vs control) as the between-subjects factor and time (T1, T2, and T3) as the within-subjects factor. The results are presented in Table 2.

**Table 2.** Mixed-design ANOVA results for intervention effects on mental health outcomes.

Outcome		*F* test (*df*)	*P* value	*η*²_p_
CES-D^a^				
Group		0.08 (1, 98)	.78	<.001
Time		7.18 (2, 196)	<.001	.07
Group × time		8.63 (2, 196)	<.001	.08
GAD-7^b^				
Group		0.74 (1, 98)	.39	.008
Time		0.70 (2, 196)	.50	.007
Group × time		1.31 (2, 196)	.27	.01
UCLA Loneliness Scale				
Group		0.26 (1, 98)	.61	.003
Time		11.13 (2, 196)	<.001	.10
Group × time		5.57 (2, 196)	.004	.05

a CES-D: Center for Epidemiologic Studies Depression Scale.

b GAD-7: Generalized Anxiety Disorder-7.

Significant group × time interactions were found for CES-D (*F*_2, 196_=8.63; *P*<.001; *η*²_p_=.08) and UCLA Loneliness Scale scores (*F*_2,196_=5.57; *P*=.004; *η*²_p_=.05), indicating differential changes in depression and loneliness between the intervention and waitlist control groups over time. These effect sizes represent medium effects according to Cohen benchmarks. No significant interaction effect was found for GAD-7 scores (*F*_2,196_=1.31; *P*=.28; *η*²_p_=.01).

For CES-D scores, post hoc comparisons with Bonferroni correction revealed that the intervention group showed significant reductions from baseline to post-intervention (T1 to T3: *t*_49_=3.85; *P*<.001; *P_adj_*=.003), as well as from midintervention to post-intervention (T2 to T3: *t*_49_=2.94; *P*=.025; *P_adj_*=.026). In contrast, the waitlist control group showed no significant change from baseline to postintervention (all *P_adj_>*.05).

Similarly, for UCLA Loneliness scores, the intervention group demonstrated a significant reduction from baseline to postintervention (T1 to T3: *t*_49_=4.28; *P*<.001; *P_adj_*<.001) and from midintervention to postintervention (T2 to T3: *t*_49_=3.44; *P*=.001; *P_adj_*=.01). No significant changes were observed in the waitlist control group (all *P_adj_>*.05).

To quantify the magnitude of these effects, we calculated Cohen *d* for the change scores (T1 to T3) between the intervention and waitlist control groups. For CES-D, this analysis yielded Cohen *d*=0.71 (95% CI 0.30‐1.17), and for UCLA Loneliness Scale, Cohen *d*=0.60 (95% CI 0.20‐1.00), representing medium to large effects. These effect sizes based on change scores better reflect the differential patterns of improvement indicated by the significant group×time interactions.

**Figure 3.**
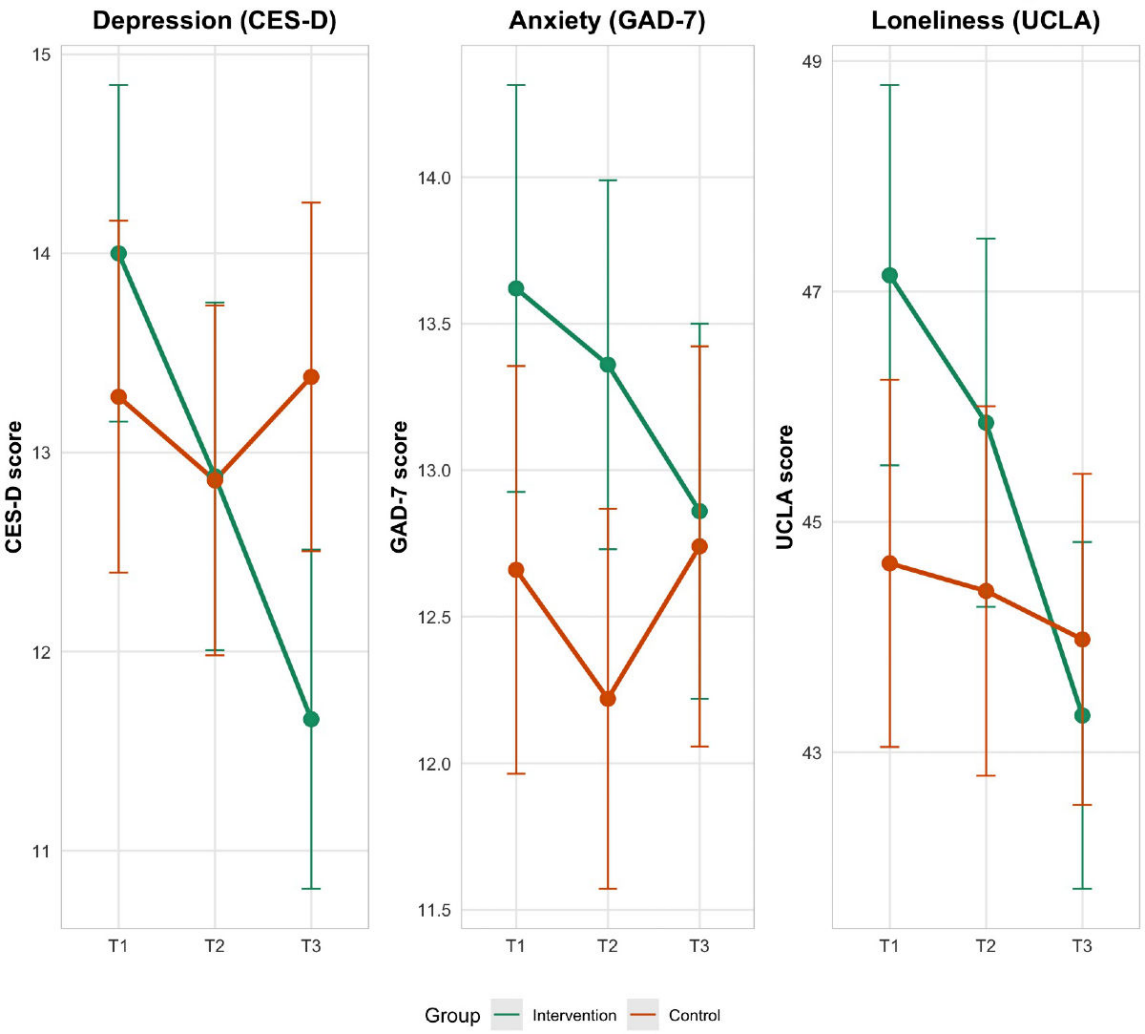
Mean scores for the CES-D, GAD-7, and UCLA Loneliness Scale across time points for the intervention and waitlist control groups. CES-D: Center for Epidemiologic Studies Depression Scale; GAD-7: Generalized Anxiety Disorder-7 scale.

Figure 3 illustrates the mean scores for CES-D, GAD-7, and UCLA Loneliness Scale across time points for the intervention and waitlist control groups.

The plots in Figure 3 illustrate the differential changes in CES-D and UCLA Loneliness Scale scores between the intervention and waitlist control groups over time. For both outcomes, the intervention group (represented by the green line) shows a steeper decline in scores compared to the waitlist control group (represented by the orange line), suggesting a greater reduction in depressive symptoms and loneliness.

### Moderation Analysis

To examine the potential moderating role of financial stress on the intervention’s effectiveness, a moderation analysis was first conducted on the entire sample using mixed linear models. The models included financial stress, time, intervention, and their interactions as fixed effects, and subject as a random effect.

For CES-D scores, the analysis revealed significant main effects of time (*F*_2,192_=6.96, *P*=.001) and financial stress (*F*_1,96_=12.96; *P*<.001), as well as significant Group×Time (*F*_2,192_=7.92; *P*<.001) and Time×Financial stress (*F*_2,192_=5.90; *P*=.003) interactions. Most importantly, a marginally significant 3-way interaction between group, time, and financial stress was observed (*F*_2,192_=2.86; *P*=.06), suggesting that the effectiveness of the intervention on depressive symptoms might vary based on participants’ financial stress levels. A post hoc power analysis indicated that with our sample size of 100 participants, α set at .05, and the corresponding effect size (*f*=0.17), the achieved power was 96.7% (see Figure S2 in Multimedia Appendix 1). This suggests adequate power for detecting the interaction effect in our primary analysis.

For UCLA Loneliness Scale scores, significant main effects were found for time (*F*_2,192_=10.54; *P*<.001) and financial stress (*F*_1,96_=23.08; *P*<.001). A significant Group×Time interaction was also observed (*F*_2,192_=5.19; *P*=.006), indicating differential changes in loneliness between the intervention and waitlist control groups over time. However, the 3-way interaction between group, time, and financial stress did not reach statistical significance (*F*_2,192_=2.32; *P*=.101), suggesting that the intervention’s effect on loneliness was not significantly moderated by financial stress.

To further explore potential differential effects based on financial stress levels, we conducted exploratory subgroup analyses for depression and loneliness. Participants in the intervention group were divided into high (n=27) and low (n=23) financial stress subgroups based on a median split of baseline PIFS scores (median=51). Separate repeated measures ANOVAs were conducted for each subgroup. The results are presented in Table 3.

For the high financial stress, significant time effects were found for CES-D (*F*_2,52_=11.56, *P*<.001, *η*²_p_=.31) and UCLA Loneliness Scale scores (*F*_2,52_=11.18, *P*<.001, *η*²_p_=.30), representing large effects. In contrast, for the low financial stress subgroup (n=23), no significant time effects were found for CES-D (*F*_2,44_=1.22, *P*=.31, *η*²_p_=.05) and only a marginally significant effect was observed for UCLA Loneliness in this subgroup (*F*_2,44_=3.44, *P*=.04, *η*²_p_=.14) with a substantially smaller effect size.

These results suggest that the intervention was particularly effective for participants with high financial stress, demonstrating large effects on both depression and loneliness in this subgroup, while showing limited or no significant effects for those with lower financial stress.

**Table 3.** Repeated measures ANOVA results for high and low financial stress subgroups.

Outcome		Effect	*F* test (*df*)	*P* value	*η*²_p_
CES-D^a^					
High stress		Time	11.56 (2, 52)	<.001	.31
Low stress		Time	1.22 (2, 44)	.31	.005
UCLA Loneliness Scale					
High stress		Time	11.18 (2, 52)	<.001	.30
Low stress		Time	3.44 (2, 44)	.04	.14

a CES-D: Center for Epidemiologic Studies Depression Scale.

**Figure 4.**
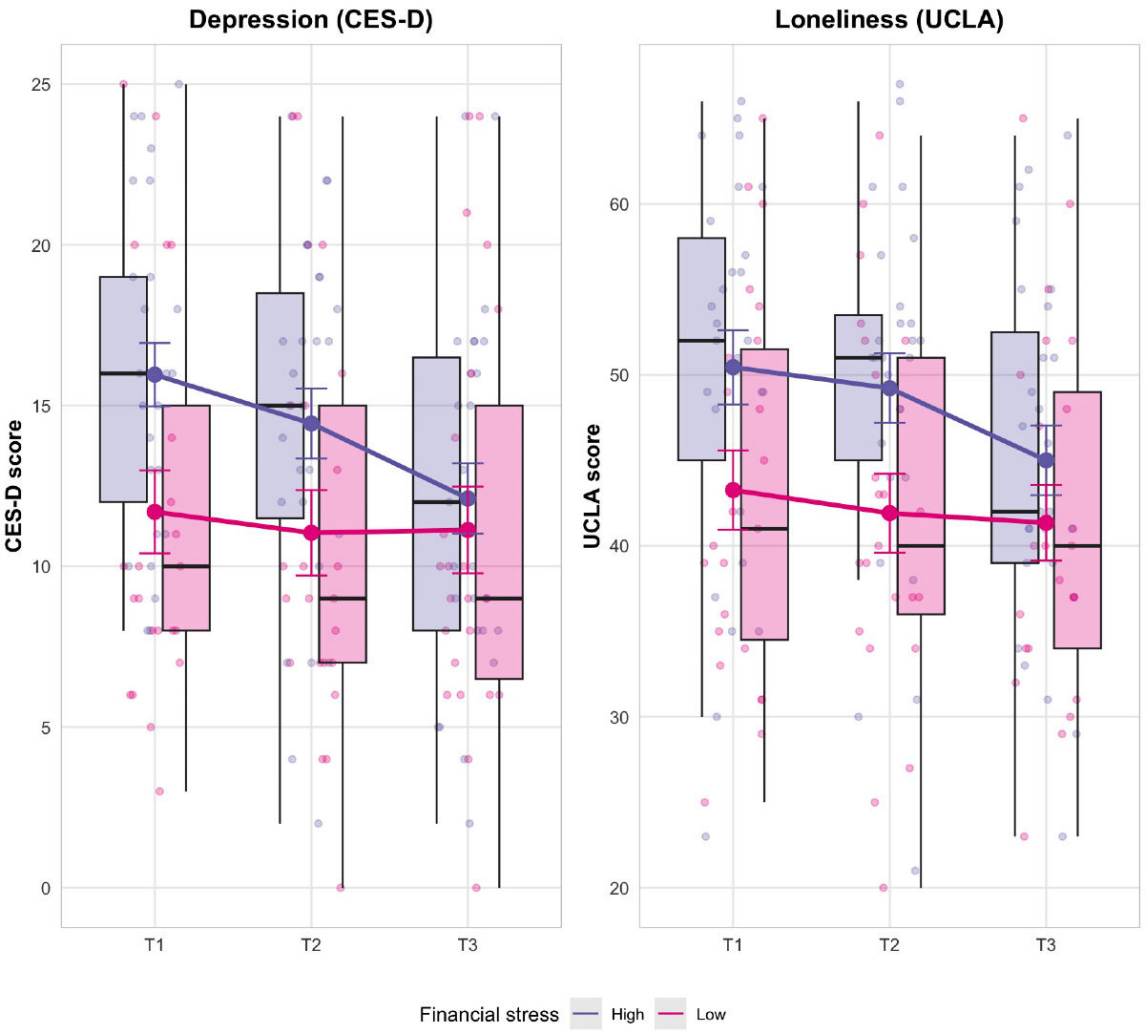
Boxplot for CES-D and UCLA Loneliness Scale across time points for high and low financial stress subgroups in the intervention group. CES-D: Center for Epidemiologic Studies Depression Scale.

Figure 4 illustrates these differential patterns, with more pronounced reductions in both depression and loneliness scores among participants with high financial stress compared to those with low financial stress. For both outcomes, participants with high financial stress show a steeper decline in scores over time, indicating greater improvements in depressive symptoms and loneliness. These visualizations provide additional support for the differential effectiveness of the intervention based on participants’ level of financial stress, with those experiencing high financial stress benefiting more from the intervention in terms of reducing depressive symptoms and loneliness.

## Discussion

### Summary of Key Findings

This study demonstrated the efficacy of a culturally adapted, CBT-based AI chatbot intervention in reducing depression and loneliness among Chinese university students, with more pronounced effects observed in students experiencing higher levels of financial stress. The intervention significantly reduced depressive symptoms and feelings of loneliness compared to a waitlist control condition, with moderate to large effect sizes (T1 to T3: Cohen *d*=0.71 and 0.60, respectively). Notably, the chatbot did not produce significant improvements in anxiety symptoms, suggesting differential effectiveness across mental health domains. Furthermore, exploratory analyses revealed that students with high financial stress benefited substantially more from the intervention than those with lower financial stress, highlighting the potential usability of this approach for addressing mental health disparities related to economic factors.

### Differential Effectiveness for Depression, Loneliness, and Anxiety

The significant reduction in depressive symptoms and loneliness among participants who engaged with the chatbot is consistent with previous studies on the effectiveness of AI-driven interventions for mental health. For example, a randomized trial of a CBT-based conversational agent (Woebot; Woebot Health) reported significant decreases in depression among young adults [25]. A scoping review likewise noted that several chatbot studies report lower loneliness, attributing this to enhanced perceived social support and engagement [24].

However, we found that the intervention did not significantly improve anxiety symptoms, which warrants further consideration, though it aligns with patterns observed in other digital mental health research. Meta-analytic data show systematically larger and more reliable effects for depression (Hedges *g*=0.56, 95% CI 0.38‐0.74) than anxiety (Hedges *g*=0.45, 95% CI 0.30‐0.6) across smartphone-based interventions [43].

Several factors may explain this differential effectiveness. First, anxiety disorders are characterized by physiological arousal components (eg, increased heart rate, muscle tension, and hypervigilance), which may be less responsive to purely cognitive-behavioral text-based interventions without complementary somatic management techniques or in-person guidance. While our chatbot included some relaxation exercises, these may have been insufficient to address the physiological aspects of anxiety effectively through a digital-only medium.

Second, the content of our chatbot focused more heavily on cognitive restructuring, targeting negative thought patterns about the self and future (which are central to depression) and behavioral activation to increase social connection (addressing loneliness), while potentially giving less attention to anxiety-specific techniques such as systematic desensitization or detailed safety behavior reduction. This content imbalance is consistent with the finding that many digital interventions emphasize depression-focused content over anxiety-specific components [18].

Third, anxiety disorders often feature avoidance behaviors that may require more intensive clinical intervention and monitoring to address effectively. Chatbot interactions may be less effective at detecting subtle avoidance patterns compared to in-person therapy. This aligns with findings from a meta-analysis, which reported stronger effects of digital interventions for depression compared to anxiety disorders, particularly when human guidance was minimal [59].

Fourth, the temporal dynamics of symptom improvement may differ between depression and anxiety. In symptom-trajectory studies, improvements in anxiety symptoms often emerge later in treatment compared to depressive symptoms. The study found that technology-based interventions for anxiety typically required a longer duration to demonstrate substantial effects [60]. Our relatively brief 7-day intervention period may have been sufficient to initiate changes in depressive symptoms but too short to observe meaningful improvements in anxiety.

Finally, cultural factors may have influenced the differential responsiveness of depression versus anxiety symptoms. In Chinese cultural contexts, expressions of depression may be more acceptable to acknowledge and address than anxiety, which is sometimes normalized as a necessary component of academic and professional achievement [32]. Cultural adaptations that more specifically target culturally shaped manifestations of anxiety might be necessary to improve the effectiveness of the intervention for anxiety symptoms in this population [36].

### The Moderating Role of Financial Stress

The exploratory subgroup analyses revealed that students experiencing high financial stress showed significant improvements in depression and loneliness, while those with low financial stress demonstrated more modest changes. This pattern, though based on exploratory analyses given the marginally significant 3-way interaction, is consistent with emerging research on the relationship between financial stress and mental health interventions.

Students with high financial stress may face additional challenges, which make them more receptive to intervention. Longitudinal evidence shows that financial difficulties predict subsequent mental health problems in university students through increased stress and reduced coping resources [10]. Our chatbot specifically addressed financial concerns through dedicated modules on cognitive restructuring for financial worries and problem-solving for financial challenges, making it particularly relevant for financially stressed students.

Financial barriers often prevent students from accessing traditional mental health services. A review found that cost concerns were among the top barriers to help-seeking among university students, with those experiencing financial hardship particularly unlikely to access in-person services [61]. The chatbot’s accessibility may have been especially valuable for these students, offering support without a financial burden. This aligns with a recent work demonstrating that digital interventions can help bridge the treatment gap for economically disadvantaged students [62].

Furthermore, the anonymity of digital interventions may be particularly important for students concerned about the stigma of help-seeking. A meta-review found that stigma concerns were heightened among individuals from lower socioeconomic backgrounds, who reported greater fears of judgment when seeking mental health support [63]. Our chatbot’s private, anonymous nature may have circumvented these concerns.

This study found that financial stress–moderated intervention effectiveness adds to emerging evidence that economic factors play a crucial role in treatment response. For instance, a web-based CBT program (“Space from Money Worries”) improved mental health and financial well-being outcomes most in participants reporting severe money concerns [64]. This suggests that comprehensive approaches that consider socioeconomic factors may enhance intervention effectiveness for vulnerable populations.

### Theoretical and Practical Implications

This study has several theoretical and practical implications. From a theoretical perspective, it supports the feasibility of adapting evidence-based CBT principles for delivery through AI chatbots, extending the reach of these interventions to populations who may not access traditional services. The results align with the unified protocol model of transdiagnostic CBT [65], which suggests that core therapeutic elements can effectively address common underlying processes across different psychological symptoms.

The differential effectiveness for various mental health outcomes highlights the importance of considering the specific mechanisms of change for different psychological symptoms and adapting digital interventions accordingly. This idea echoes the behavioral-intervention-technology model, which emphasizes the need to match intervention components to specific therapeutic targets and mechanisms [66].

Practically, this study supports the integration of AI-driven interventions into existing university mental health support systems. The findings suggest that such interventions can be particularly beneficial for financially stressed students, providing a way to overcome barriers to traditional mental health services. Universities could consider implementing similar chatbot interventions as part of a stepped care approach, potentially using them as an initial intervention or as a supplement to traditional services, as recommended by Lattie et al [20] in their framework for digital mental health implementation in higher education settings.

Furthermore, the cultural adaptation process demonstrated in this study provides a framework for developing culturally sensitive digital mental health interventions. The incorporation of cultural elements, such as collectivistic values, indirect communication styles, and culturally relevant metaphors, may have enhanced the acceptability and effectiveness of the intervention for Chinese students. This approach aligns with the framework for cultural adaptation of psychological interventions and could be applied to adapt digital interventions for other cultural contexts, potentially improving their global applicability.

### Limitations and Future Directions

Our sample size (N=100) was adequate for detecting primary effects according to our a priori power analysis (which determined a minimum sample of 52 participants was needed), but limited the power of subgroup and moderation analyses. The exploratory nature of our subgroup analyses, with relatively small sample sizes in each financial stress category (27 high stress and 23 low stress in the intervention group), limits the robustness of our moderation findings. Future research with larger samples specifically powered for moderation analyses is needed to confirm the differential effectiveness of the intervention based on financial stress levels.

In addition, our exclusion criteria (current psychotherapy, psychiatric-medication use, or a history of severe mental illness) restrict generalizability to the wider student population, where complex mental health needs are common. Although these criteria were necessary to isolate intervention effects and protect participants in this initial evaluation, future work should test the chatbot as an adjunct to conventional treatments in more clinically diverse samples, in keeping with recommendations that digital tools complement rather than replace routine care [67].

The use of a waitlist control group, while providing a baseline for comparison, has inherent limitations recognized in digital mental health research [45]. Waitlist controls may not account for nonspecific factors such as expectancy effects or attention. Participants in waitlist conditions may also postpone seeking help or making changes while waiting for the intervention, potentially exaggerating between-group differences. Future studies should consider active control conditions, such as psychoeducational resources or attention-matched control interventions, to better isolate the specific effects of the chatbot’s therapeutic components.

Our reliance on self-report measures may have introduced reporting biases. Cultural factors might influence how Chinese students report mental health symptoms, potentially affecting the accuracy of our assessments. Chinese students, for example, tend to emphasize somatic rather than psychological complaints, which may have affected our measurement of mental health outcomes. Future studies should incorporate multiple assessment methods, including clinician-rated measures, behavioral indicators, or physiological markers, to provide a more comprehensive evaluation of intervention effects.

The relatively brief 7-day intervention period may have been insufficient to produce meaningful changes in anxiety symptoms or to establish lasting improvements in depression and loneliness. Meta-analytic evidence indicates stronger effects for digital CBT delivered over a long period [68]. Future studies should investigate longer intervention durations and include follow-up assessments to evaluate the sustainability of effects over time.

### Implementation Considerations

The high adherence rate observed in our study (47/50, 94% in the intervention group) exceeds typical rates reported in digital intervention research, where adherence rates of 40%‐60% are common. While this speaks to the engaging nature of the chatbot, it may have been influenced by the monetary compensation provided to participants, particularly relevant for financially stressed students. In real-world implementations without financial incentives, adherence rates might be lower. Future research should explore sustainable engagement strategies that do not rely on monetary compensation, such as gamification elements, personalized messaging, or integration with existing university systems, which have been proposed [69] and should be explored.

While our chatbot incorporated several advanced features, it still had technical limitations. The rule-based and template-based responses (comprising 90% of interactions) may not fully capture the nuanced needs of users compared to more sophisticated AI systems. As natural language processing technology advances, future iterations could incorporate more adaptive and personalized interaction capabilities while maintaining clinical safety and appropriateness.

Finally, despite substantial efforts we made to culturally adapt the chatbot, there may be deeper cultural nuances that were not fully addressed. Future research could use more comprehensive cultural adaptation frameworks and involve larger, more diverse groups of cultural consultants and end users in the development process to enhance cultural relevance and acceptability.

### Conclusions

This study demonstrated that a culturally adapted, CBT-based AI chatbot can effectively reduce depression and loneliness symptoms among Chinese university students, with particularly strong effects for those experiencing high financial stress. These findings highlight the potential of digital mental health interventions to address mental health disparities related to economic factors and expand access to support for underserved populations.

The differential effectiveness across mental health domains, with significant improvements in depression and loneliness but not anxiety, underscores the importance of tailoring digital interventions to specific psychological mechanisms and cultural contexts. Future developments should incorporate stronger anxiety-specific components and longer intervention periods to potentially enhance effects across a broader range of mental health outcomes.

As universities worldwide face increasing demand for mental health services amid resource constraints, culturally adapted digital interventions offer a promising approach to extend support to students who might otherwise go untreated. The moderating effect of financial stress suggests that such interventions may be particularly valuable for economically disadvantaged students, potentially helping to address socioeconomic disparities in mental health treatment access and outcomes.

Future research should focus on refining these interventions to address anxiety more effectively, investigating their long-term effects, and exploring their implementation in diverse real-world settings. In addition, studies examining the specific mechanisms through which financial stress impacts mental health and how digital interventions can best address these mechanisms would further enhance our understanding and improve intervention design for vulnerable student populations.

## Supplementary material

10.2196/63806 Multimedia Appendix 1 Power analysis.

10.2196/63806 Multimedia Appendix 2 The detailed cognitive behavioral therapy (CBT) module structure, cultural adaptation framework, validation metrics, and sample chatbot dialogue examples.

10.2196/63806 Checklist 1 CONSORT-EHEALTH Checklist (V 1.6.1).

## Abbreviations

AI: artificial intelligence
CBT: cognitive behavioral therapy
CES-D: Center for Epidemiologic Studies Depression Scale
CONSORT: Consolidated Standards of Reporting Trials
GAD-7: Generalized Anxiety Disorder-7 scale
PIFS: Psychological Inventory of Financial Scarcity
