# Supplementary material for: Effect of a Cognitive Behavioral Therapy–Based AI Chatbot on Depression and Loneliness in Chinese University Students: Randomized Controlled Trial With Financial Stress Moderation
Source: JMIR Mhealth Uhealth. 2025 Aug 29;13:e63806. doi: 10.2196/63806 (PMC12396778; doi:10.2196/63806)

**Supplementary: Power Analysis for Primary and Moderation Analyses**

**A priori Power Analysis**

We conducted a priori power analysis using G*Power 3.1 to determine the necessary sample size for our primary analysis (2×3 mixed ANOVA). The analysis was performed using the following steps in G*Power:

1. Test family: F tests
2. Statistical test: ANOVA: Repeated measures, within-between interaction
3. Type of power analysis: A priori
4. Input parameters:
   - Effect size f: 0.20 (small-to-medium effect)
   - α err prob: 0.05
   - Power (1-β err prob): 0.80
   - Number of groups: 2 (intervention and waitlist control)
   - Number of measurements: 3 (baseline, mid-intervention, post-intervention)
   - Corr among rep measures: 0.5 (moderate correlation)
   - Nonsphericity correction ε: 1

The analysis indicated that a total sample size of 42 participants (21 per group) would be required to detect a small-to-medium effect size (f = 0.20) with 80% power. Adjusting for an anticipated 31% attrition rate (the weighted average for internet-based treatment programs according to Melville et al., 2010), the adjusted total sample size needed was 60 participants.

To ensure robust statistical power even with potential higher-than-expected attrition, we aimed for a sample of 100 participants (50 per group), which exceeded the minimum requirement substantially. This larger sample size enhanced our ability to detect the hypothesized effects and provided additional power for exploratory analyses.

**Figure S1.** A priori Power Analysis


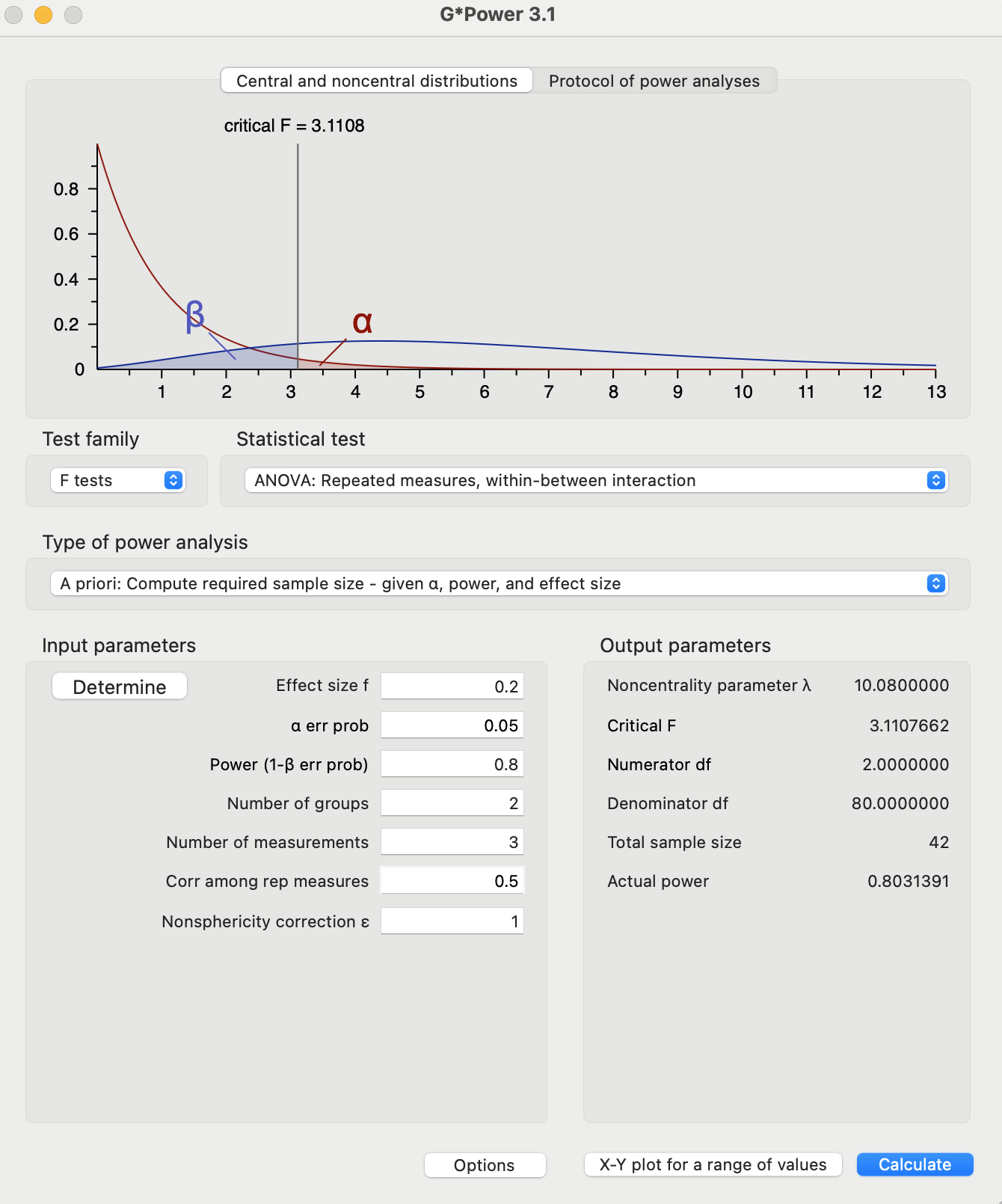


**Post hoc Power Analysis for Moderation Effects**

For the moderation analysis (three-way interaction between Group × Time × Financial Stress), we conducted a post hoc power analysis using the following steps in G*Power:

1. Test family: F tests
2. Statistical test: ANOVA: Repeated measures, within-between interaction
3. Type of power analysis: Post hoc
4. Input parameters:
   - Effect size f: 0.17 (calculated from the observed η²ₚ = 0.029 using the formula f = √[η²ₚ/(1-η²ₚ)])
   - α err prob: 0.05
   - Total sample size: 100
   - Number of groups: 2
   - Number of measurements: 3
   - Corr among rep measures: 0.5
   - Nonsphericity correction ε: 1

The analysis yielded a power of 96.7%, indicating that our sample size was sufficient to detect the observed effect size for the three-way interaction with high reliability. This high power reflects our larger-than-necessary sample size (N=100) relative to the minimum required by our a priori calculation (N=42).

**Figure S2.** Post hoc Power Analysis for Moderation Effects


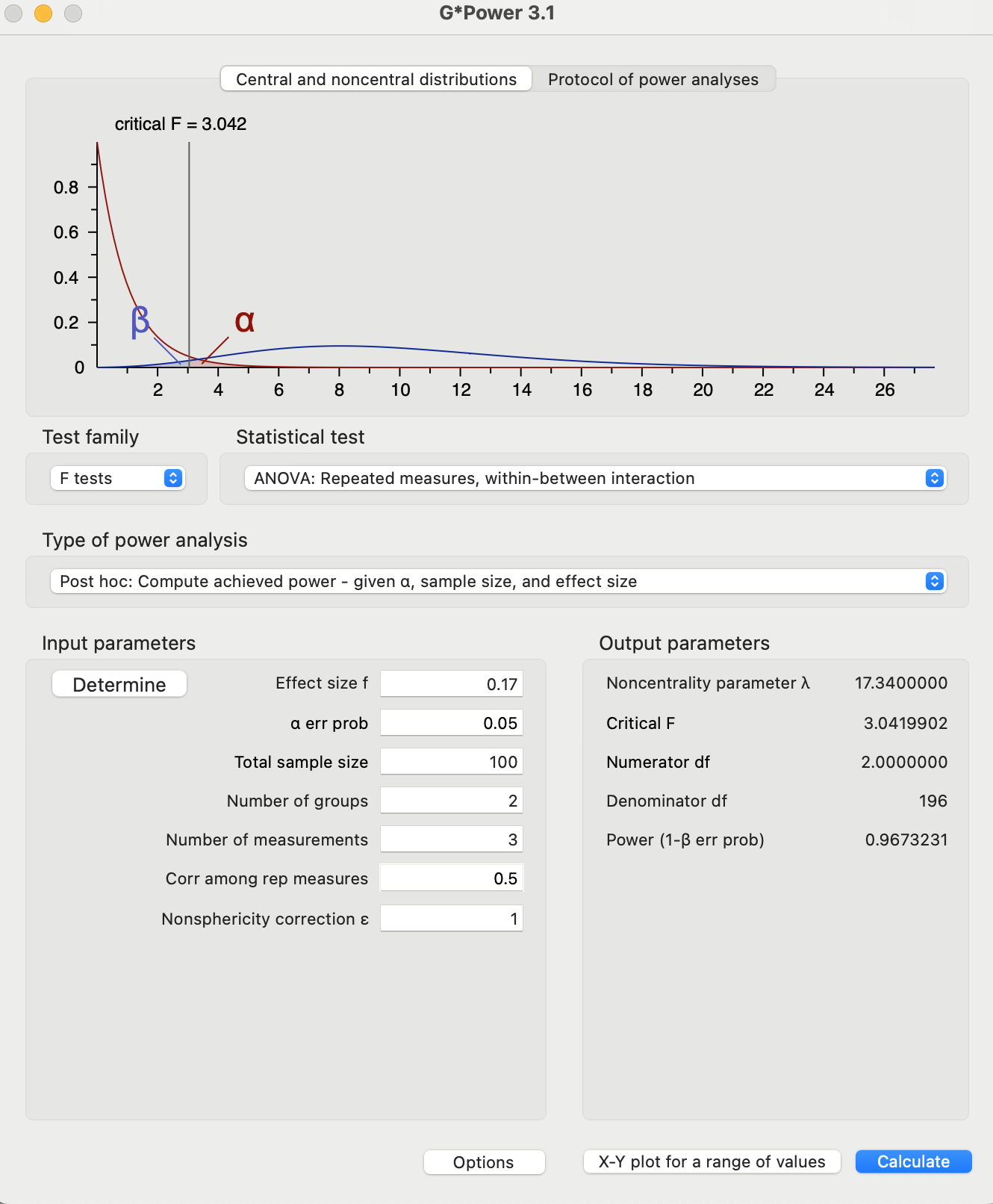

Supplement: Multimedia Appendix 1 [file mhealth-v13-e63806-s001.docx]
