# Supplementary material for: Effect of a Cognitive Behavioral Therapy–Based AI Chatbot on Depression and Loneliness in Chinese University Students: Randomized Controlled Trial With Financial Stress Moderation
Source: JMIR Mhealth Uhealth. 2025 Aug 29;13:e63806. doi: 10.2196/63806 (PMC12396778; doi:10.2196/63806)
Supplement: Multimedia Appendix 2 [file mhealth-v13-e63806-s002.docx]

**Table S1:** Seven-Day CBT Module Structure

| **Day** | **Module** | **Core CBT Components** | **Key Therapeutic Techniques** | **Cultural Adaptations** |
| --- | --- | --- | --- | --- |
| 1 | Introduction to CBT principles | Psychoeducation; Cognitive model | Thought-emotion-behavior connections; Self-monitoring | Integration of Chinese philosophical concepts of balance; Use of culturally relevant examples |
| 2 | Coping with campus-related stressors | Cognitive restructuring; Stress management | Identifying cognitive distortions; Developing coping statements | Addressing academic pressure within Chinese educational context; Respect for authority while developing assertiveness |
| 3 | Adapting to new environments | Behavioral activation; Exposure techniques | Gradual exposure to new situations; Building routines | Balancing individual adaptation with family connections; Addressing homesickness in collectivistic context |
| 4 | Managing anxiety | Cognitive restructuring; Relaxation training | Identifying catastrophic thinking; Breathing techniques | Incorporating traditional Chinese relaxation practices; Addressing somatic expressions of anxiety common in Chinese populations |
| 5 | Financial stress management | Problem-solving; Cognitive reframing | Financial planning; Addressing money-related thoughts | Addressing filial obligation and guilt about family financial sacrifice; Face-saving strategies for financial limitations |
| 6 | Emotion regulation and mood improvement | Behavioral activation; Mindfulness | Pleasant activity scheduling; Present-moment awareness | Low-cost activities suitable for financial constraints; Mindfulness practices with Chinese cultural elements |
| 7 | Building resilience and relapse prevention | Consolidation; Maintenance planning | Skill review; Future planning | Integration of collective support resources; Long-term plans respecting family obligations and personal growth |

**Table S2:** Cultural Adaptation Based on Bernal et al.’s Framework

| **Adaptation Dimension** | **Definition** | **Standard CBT Approach** | **Cultural Adaptation for Chinese Context** | **Example from Chatbot Content** |
| --- | --- | --- | --- | --- |
| Language | Linguistic elements including idioms | Direct expression of emotions | Indirect expression patterns; Use of somatic descriptions | “You might notice tension in your shoulders when thinking about finances” instead of “You feel anxious about money” |
| Persons | Role of cultural factors in therapeutic relationship | Egalitarian therapist-client relationship | Incorporation of respectful guidance reflecting teacher-student dynamics valued in Chinese culture | More directive guidance in problem-solving while maintaining warmth and empathy |
| Metaphors | Cultural symbols and concepts | Western metaphors (e.g., “fighting” depression) | Traditional Chinese metaphors and idioms | Using “finding balance between mountains and water” to explain emotional regulation |
| Content | Cultural knowledge integrated into materials | Universal examples and scenarios | Specific content addressing Chinese university experiences | Scenarios about managing family expectations during university transitions |
| Concepts | Theoretical constructs underlying treatment | Individual-focused psychological theories | Integration with Chinese cultural concepts | Incorporating “face” and “harmony” into cognitive restructuring exercises |
| Goals | Purpose and aims of treatment | Emphasis on personal autonomy and symptom reduction | Balance between individual wellbeing and social harmony | Setting goals that benefit both personal health and family relationships |
| Methods | Procedures and processes of treatment | Direct challenging of thoughts; individual homework | More indirect approaches; inclusion of family where appropriate | Using third-person examples and stories to introduce alternative perspectives |
| Context | Broader social context considerations | Generic therapeutic settings | Specific adaptation to Chinese university environment | Addressing academic pressure in the context of China’s educational system and job market |

**Table S3: Validation Results and Metrics**

| **Validation Component** | **Evaluators/Participants** | **Assessment Area** | **Detailed Ratings** | **Mean Score** |
| --- | --- | --- | --- | --- |
| **Content Validation** |  |  |  |  |
|  | Clinical Psychologist 1 | Adherence to CBT principles | 4.3/5 |  |
|  | Clinical Psychologist 2 | Adherence to CBT principles | 4.0/5 |  |
|  | Clinical Psychologist 3 | Adherence to CBT principles | 4.3/5 | 4.2/5 |
|  | Clinical Psychologist 1 | Clinical appropriateness | 4.5/5 |  |
|  | Clinical Psychologist 2 | Clinical appropriateness | 4.1/5 |  |
|  | Clinical Psychologist 3 | Clinical appropriateness | 4.3/5 | 4.3/5 |
|  | Clinical Psychologist 1 | Alignment with student needs | 4.2/5 |  |
|  | Clinical Psychologist 2 | Alignment with student needs | 3.9/5 |  |
|  | Clinical Psychologist 3 | Alignment with student needs | 4.2/5 | 4.1/5 |
| **Cultural Validation** |  |  |  |  |
|  | Cultural Expert 1 | Cultural relevance | 4.2/5 |  |
|  | Cultural Expert 2 | Cultural relevance | 4.0/5 | 4.1/5 |
|  | Cultural Expert 1 | Linguistic appropriateness | 4.4/5 |  |
|  | Cultural Expert 2 | Linguistic appropriateness | 4.2/5 | 4.3/5 |
|  | Cultural Expert 1 | Contextual fit | 3.7/5 |  |
|  | Cultural Expert 2 | Contextual fit | 4.1/5 | 3.9/5 |
| **User Testing** |  |  |  |  |
|  | Student 1 | System Usability Scale | 84/100 |  |
|  | Student 2 | System Usability Scale | 79/100 |  |
|  | Student 3 | System Usability Scale | 86/100 |  |
|  | Student 4 | System Usability Scale | 80/100 |  |
|  | Student 5 | System Usability Scale | 76/100 |  |
|  | Student 6 | System Usability Scale | 85/100 |  |
|  | Student 7 | System Usability Scale | 89/100 |  |
|  | Student 8 | System Usability Scale | 81/100 |  |
|  | Student 9 | System Usability Scale | 83/100 |  |
|  | Student 10 | System Usability Scale | 80/100 | 82.3/100 |
|  | All Students (n=10) | Comprehension rating | Range: 3.9-4.7/5 | 4.4/5 |

**Table S4:** Sample Chatbot Dialogue Examples

| **Topic** | **User Input** | **Chatbot Response** | **CBT Technique** | **Cultural Adaptation Element** |
| --- | --- | --- | --- | --- |
| Financial Stress | “I feel ashamed that my classmates can buy new things but I have to be so careful with money.” | “Many students compare their financial situation with others. This comparison can create difficult feelings. Instead of focusing on what others have, maybe we can explore what matters most to you personally. What values guide your financial decisions?” | Cognitive restructuring (addressing comparison thoughts) | Addressing “face” concerns while respecting collectivistic context |
| Adapting to New Environment | “I miss home and feel out of place in this new city.” | “Moving to a new place can be challenging. Many students feel a sense of displacement (背井离乡). Perhaps we can identify some small steps to help you build connection to this new place while honoring your connection to home.” | Behavioral activation; Graded exposure | Using cultural idioms; Balancing adaptation with maintaining family ties |
| Anxiety Management | “I’m constantly worried about getting sick during the pandemic.” | “It’s natural to be concerned about health, especially during uncertain times. Many people share this concern. Let's explore which of these worries are within your control and which ones might be consuming energy without helping you stay safe.” | Cognitive restructuring; Problem-solving | Acknowledging collective experience; Practical focus aligned with Chinese pragmatic approach |
| Campus Bullying | “Some classmates exclude me from study groups because I’m from a rural area.” | “I’m sorry to hear about this experience. Such exclusion can be hurtful. In Chinese university culture, study groups are important both socially and academically. Let’s think about some ways you might navigate this situation while preserving your dignity.” | Assertion training; Cognitive reframing | Acknowledging regional discrimination issues in Chinese educational context |
